# Curcumin (1,7-bis(4-hydroxy-3-methoxyphenyl)-1, 6-heptadiene-3,5-dione) Blocks the Chemotaxis of Neutrophils by Inhibiting Signal Transduction through IL-8 Receptors

**DOI:** 10.1155/2007/10767

**Published:** 2007-06-28

**Authors:** Masafumi Takahashi, Takatoshi Ishiko, Hidenobu Kamohara, Hideaki Hidaka, Osamu Ikeda, Michio Ogawa, Hideo Baba

**Affiliations:** ^1^Department of Gastroenterological Surgery, Graduate School of Medical Sciences, Kumamoto University, Honjo 1-1-1, Kumamoto 860-8556, Japan; ^2^Department of Surgery II, Faculty of Medicine, University of Miyazaki, Miyazaki-shi, Miyazaki 889-2192, Japan; ^3^Department of Surgery, Kumamoto Rousai Hospital, Yatsushiro 866-8533, Japan

## Abstract

We investigated the impact of curcumin on neutrophils. Chemotactic activity via human recombinant IL-8 (hrIL-8) was significantly inhibited by curcumin. Curcumin reduced calcium ion flow induced by internalization of the IL-8 receptor. We analyzed flow cytometry to evaluate the status of the IL-8 receptor after curcumin treatment. The change in the distribution of receptors intracellularly and on the cell surface suggested that curcumin may affect the receptor trafficking pathway intracellulary. Rab11 is a low molecular weight G protein associated with the CXCR recycling pathway. Following curcumin treatment, immunoprecipitation studies showed that the IL-8 receptor was associated with larger amounts of active Rab11 than that in control cells. These data suggest that curcumin induces the stacking of the Rab11 vesicle complex with CXCR1 and CXCR2 in the endocytic pathway. The mechanism for antiinflammatory response by curcumin may involve unique regulation of the Rab11 trafficking molecule in recycling of IL-8 receptors.

## 1. INTRODUCTION

Curcumin (17-bis (4-hydroxy-3-methoxyphenyl)-1,6-hepta-diene-3,5-dione) extracted from *Curcuma longa* L. is generally used as a spice and as a coloring agent in food [1, 2]. Curcumin is also used in traditional medicine to treat the inflammatory diseases [3].

Curcumin has anti-inflammatory and antioxidant effects and downregulates chemokine expression in inflammatory cells [1, 4, 5]. Curcumin affects kinase reactions, such as those of MAP kinase, and PKC, c-Jun/AP-1 [6, 7]. Furthermore, several researchers, including our group, have reported that curcumin blocks NF-kB activity during transcription [8, 9]. The molecular mechanism of NF-kB inhibition involves the blocking of IkB phosphorylation in a human nontransforming epithelial cell line [9, 10]. Although the inhibitory effect of curcumin on inflammatory responses is well known [11, 12], the contribution of curcumin to the signal transduction pathway in the proinflammatory cytokine response remains unclear.

IL-8 belongs to the CXC chemokine subgroup. It is a chemotactic cytokine that activates and elicits the migration of leukocyte [13]. IL-8 affects the functions of human neutrophils, including enhanced chemotaxis, enzyme release, and expression of surface adhesion molecules. IL-8 stimulates neutrophils via specific chemokine receptors, namely, the IL-8 receptors. These receptors are seven-transmembrane guanine nucleotide-binding protein-coupled receptors (GPCRs). IL-8 receptors are membrane-bound proteins expressed in large amount in primary cultured neutrophils. Humans have two high-affinity receptors for IL-8, namely, CXCR1 and CXCR2. The sequences of these receptors show 77% homology [13, 14]. Receptor-mediated signal transduction is initiated by coupling with heterotrimeric G proteins, and this results in an increase in the cytosolic free Ca^2+^ concentration due to activation by IL-8 [14, 15]. CXCR1 has limited activators, such as IL-8; however, CXCR2 has numerous activator cytokines, that is, CXC chemokines such as NAP-2 and GRO*α* [16, 17]. Recent evidence has suggested that IL-8 receptors internalize via clathrin-coated vesicle upon agonist binding [18, 19]. After the release of the agonist, the IL-8 receptors are transported from the cytoplasm to the nucleus; some of the receptors are transported back to the cell surface via the recycling endosomes. This phenomenon is termed as “recycling.” CXCR1 is recycled to the cell surface more rapidly than CXCR2 [17, 19–22]. It was reported that CXCR1 is the dominant mediator of the neutrophil chemotactic response to IL-8 [23]. Thus, CXCR1 is involved in the crucial process of neutrophil-induced inflammation.

We previously reported that IL-8 and its receptors were constitutively produced by pancreatic cancer cells. In addition, the autocrine growth effect of IL-8 is blocked by curcumin [8]. In this process, curcumin contributes not only to the inhibition of IL-8 production but also to the inhibition of signal transduction via IL-8 receptors. These data suggested that curcumin is a potent agent that blocks the IL-8 receptor-mediated neutrophil responses.

In this study, we investigated the effect of curcumin on neutrophil chemotaxis mediated by IL-8 and determined whether curcumin treatment inhibits signal transduction from the IL-8 receptor in human primary neutrophils. We designed the study in order to test the hypothesis that curcumin treatment has an effect on the endosomal trafficking pathway of IL-8 receptors.

Therefore, the endosomal vesicles that participate in the trafficking of IL-8 receptors may play an important role in the curcumin-mediated regulation. The Rab proteins Ras-related small guanosine-5′-triphosphatases (GTPases) might be essential since they are involved in the mechanisms of trafficking intracellular vesicles to the target organs [24–26]. More than 60 types of Rab proteins were found, and they play a role in regulating some of the steps involved in the intracellular trafficking of target molecules [25]. Considerable evidence showed that CXCR2 and Rab11 coexist in the recycling step of CXCR2-transfected cells [27, 28]. These data suggested that Rab11 might be a key mediator in the distribution and trafficking pathway of IL-8 receptors upon curcumin treatment.

In this study, our data showed that curcumin treatment regulated the recycling of IL-8 receptors and the amount of cell surface IL-8 receptors. This change may be closely associated with the anti-inflammatory response caused by the interference of the chemotactic activity of IL-8.

## 2. MATERIALS AND METHODS

### 2.1. Reagents

Curcumin was purchased from Sigma (St. Louis, Mo, USA). Polymorphprep was obtained from Axis-Shield (Oslo, Norway). IntraPrep permeabilization reagent was obtained from Immunotech (Marseille, France), rIL-8 from R&D Systems Inc. (minneapolis, Minn, USA), Diff-Quick from Midori Juji (Osaka, Japan), Fura Red-AM from Molecular Probes (Eugene, Ore, USA). Anti-CXCR1 mouse monoclonal IgG_1_ was purchased from Santa Cruz Biotechnologies (Santa Cruz, Calif, USA). Anti-CXCR2 mouse monoclonal IgG_1_, mouse IgG_1_, allophycocyanin (APC)-conjugated rat antimouse IgG_1_ monoclonal antibody, and purified mouse IgG_1_ monoclonal IgG standard were purchased from BD Pharmingen (San Diego, Calif, USA). Human IgG was purchased from Sigma. Cycloheximide was purchased from Wako Pure Chemical Industries Ltd. (Osaka, Japan).

### 2.2. Neutrophil isolation

Neutrophils were isolated from the peripheral venous blood of healthy human volunteers. Heparinized whole blood was layered onto Polymorphoprep (Axis-Shield PoC AS) and centrifuged at 500 x*g* for 35 minutes. The supernatant and the peripheral blood mononuclear cell (PBMC) layers were discarded. The polymorphonuclear neutrophil (PMN) layer was removed, and hyposmotic hemolysis was performed for the remaining erythrocytes. Since NH_4_Cl, a lysosomotrophic agent, may inhibit receptor trafficking in human neutrophils, the neutrophils used in these experiments were prepared without NH_4_Cl. The PMNs were resuspended in RPMI 1640 medium containing 10% fetal calf serum (FCS). The purity of the neutrophil preparations was routinely found to be greater than 95% with viability greater than 98%, as observed by trypan blue exclusion test.

### 2.3. Curcumin treatment

Curcumin was dissolved in dimethyl sulfoxide (DMSO) on the day of use, and was added to the cells at a final concentration of 0.5–100 *μ*M. The DMSO concentration was always less than 0.1% (vol/vol).

### 2.4. Trypan blue staining

Trypan blue solution (0.4%) was added to the cell suspension (final concentration, 0.2%). After treatment with curcumin (10–100 *μ*M) for 2 hours, the number of positive cells was counted in triplicate.

### 2.5. Neutrophil chemotaxsis assay

Chemotaxis was assessed by a modification of a previously described method; 24-well microchemotaxis chambers (Becton Dickinson, Cowley, UK) were used, and these were covered with polyethylene terephthalate membranes with pore size of 3 *μ*m [29, 30]. The lower wells were filled with 700 *μ*L of medium with various concentrations of recombinant human IL-8 (hrIL-8) (10 ∼ 10000 ng/mL) or medium without hrIL-8. Subsequently, 700 *μ*L of a suspension containing 1 × 10^6^ PMNs was added to each well of the upper chamber. After incubation under conditions of 90% humidity and 5% CO_2_ for 1 hour at 37°C, the cells that passed through the filter to the lower wells were stained with Diff-Quick and counted in 5 high-power fields (hpf, 40 × 10) using a calibrated grating. Neutrophil chemotactic activity was determined in triplicates and expressed as the average number of migrating neutrophils/5 hpf.

Next, to determine the effect of curcumin on neutrophil chemotaxis, the neutrophils were preincubated with various concentrations of curcumin (10 ∼ 100 *μ*M) at 37°C for 2 hours. The preincubated neutrophils were added to each well of the upper chamber and incubated for 1 hour, as described above. The cells that passed through the filter to the lower wells were stained and counted. The data obtained was expressed as described above.

### 2.6. Measurement of cytosolic calcium

The obtained neutrophils were colored yellow by curcumin that has a bimodal peak of absorption at 210 nm and 425 nm. The color causes fluorescence noise when using optical instruments. To avoid interference due to the fluorescence noise, we used Fura Red because it shows decreased red fluorescence that is not interfered by curcumin. Neutrophils (1 × 10^7^ cells/mL) were loaded with 13 *μ*M Fura Red-AM in HEPES-buffered saline (135 mM NaCl, 4.6 mM KCl, 1.2 mM MgCl_2_, 11 mM HEPES, 11 mM glucose, 1.5 mM CaCl_2_, at pH 7.4) for 90 minutes at 37°C in a CO_2_ incubator. The loaded neutrophils were then analyzed using a FACS calibur system (Beckton Dickinson, USA) for analyzing red fluorescence (640 nm) following excitation at 488 nm using an argon laser. The mean channel of fluorescence was detected by CellQuest software (Beckton Dickinson) [31, 32]. The measurements of the mean channel of fluorescence can be used for statistical comparison of the cells before and after hrIL-8 treatment. The data obtained showed differences between the mean channel value before and after IL-8 stimulation.

### 2.7. Analysis of CXCR internalization

Internalization analysis was performed as previously described [8, 15]. In brief, neutrophils were preincubated with RPMI 1640 medium containing 10% FCS supplemented with 50 *μ*M curcumin or without curcumin at 37°C for 2 hours. The cells were washed twice in PBS and once in bovine serum albumin medium (BSA medium = RPMI 1640 medium containing 1% bovine serum albumin and 25 mM HEPES). Aliquots of 5 × 10^6^ cells were then removed and supplemented with 50 ng/mL hrIL-8 diluted in BSA medium, while no hrIL-8 was added to the control tubes. The cells were incubated at 37°C for 2 hours and then incubated for an additional 10 minutes on ice. The cells were washed using the cell sorter buffer (CSB: PBS containing 1% FCS, 0.02% NaN_3_, and 25 mM HEPES) and incubated at 4°C for 15 minutes with human IgG to block the Fc receptors. They were washed twice with CSB and incubated with either monoclonal mouse anti-CXCR1 or anti-CXCR2 antibody and mouse-IgG1a as the control. Following the 15-minute incubation, the cells were washed twice in CSB, and APC-conjugated rat antimouse IgG antibody was added and incubated at 4°C for 15 minutes. Since APC emission is collected in a detector for fluorescence wavelengths between 640 nm and 680 nm, in these experiments, interference by curcumin fluorescence should be avoided.

The cells were then washed once in CSB and resuspended in PBS containing 0.02% NaN_3_. Flow cytometry was performed using the FACS calibur system. It was used to analyze 10000 live cell events.

### 2.8. Staining of both cell surface and intracytoplasmic CXCR

The neutrophils were permeabilized using the IntraPrep permeabilization reagent, including reagents 1 and 2, according to the manufacturer's instructions [33]. In brief, to stain CXCR present on the surface of the cell membrane, the neutrophils were incubated with a primary antibody at 4°C for 15 minutes, washed twice with CSB, and stained with the secondary antibody for 15 minutes. Following incubation, 100 *μ*L of IntraPrep reagent 1 was added for fixation. After vigorous vortexing, the cells were incubated for 15 minutes at room temperature. After one wash with PBS, 100 *μ*L of IntraPrep reagent 2 was added, and then the mixture was incubated for 5 minutes at room temperature for permeabilization. Next, the neutrophils were incubated with the primary antibody at 4°C for 15 minutes, and the intracellular IL-8 receptors were incubated with the secondary antibody for 15 minutes. Following these incubations, the cells were washed once with CSB and resuspended in PBS containing 1% formaldehyde. Subsequently, flow cytometry analysis was performed.

### 2.9. Western blotting

The pretreated neutrophils were lysed in a “lysis buffer” comprising 50 mM HEPES (pH 7.4), 150 mM NaCl, 1 mM EDTA, 1% Triton X-100, 10% Glycerol, 1mM NaF, 2 mM Na orthovandate, 0.1 nM phenylmethylsulfonyl fluoride, 5 *μ*g/mL leupeptin, 4.6 *μ*g/mL aprotinin, and 3.5 ng/mL Pepstatin A. Proteins in the cell lysates were separated by sodium dodecyl sulfate-polyacrylamide gel electrophoresis (SDS-PAGE) under reducing conditions and then blotted onto nitrocellulose membranes (Hybond C^+^, Amersham Pharmacia Biotech, UK). The membranes were blocked in NET buffer (50 mM Tris-HCl, pH 7.5, 150 mM NaCl, 5 mM EDTA, 0.05% Triton X-100) containing 1% gelatin and then incubated sequentially with the respective antibodies and rabbit or mouse IgG TrueBlot (eBioscience, San Diego, Calif, USA). The protein bands were visualized with an enhanced chemiluminescence (ECL) detection kit (Amersham Pharmacia Biotech).

### 2.10. Immunoprecipitation analysis

The neutrophil lysate was obtained as described above. The lysates were precleared by incubation for 1 hour with TrueBlot antimouse Ig IP beads (eBioscience). The primary antibody and TrueBlot antimouse Ig IP beads were added to the precleared lysates. The beads were washed in a washing buffer [50 mM HEPES (pH 7.4), 150 mM NaCl, 0.1% Triton X-100, 10% Glycerol] and resuspended in the SDS sample buffer. Immunoprecipitation and western blotting were performed as described above.

### 2.11. Determination of IL-8 receptors recycling on the cell surface

The procedure used to determine receptor recycling was similar to that used to determine receptor internalization. The samples were allowed to undergo a receptor recovery process that was performed as previously described [17]. In brief, internalization was induced by exposure to 1000 ng/mL hrIL-8 for 30 minutes at 37°C. In order to allow receptor recovery, the cells were washed once and resuspended in BSA medium at 37°C for the specified time periods. The neutrophils were incubated with the specified concentrations of curcumin during the IL-8 receptor recovery process. To reduce the contribution of de novo protein synthesis to receptor reappearance on the cell membrane, cell samples undergoing receptor recovery were incubated with medium containing 10 *μ*g/mL cycloheximide. Following incubation, the cells were washed and labeled at 4°C with anti-CXCR1 or anti-CXCR2 antibody and mouse IgG, as described above. The FACS calibur system was used to analyze 10000 live cell events.

### 2.12. Statistical analysis

Data are expressed as mean values ± standard error. Statistical significance was determined by Student *t* tests and correlation coefficients. A *P* value less than .05 was considered significant.

## 3. RESULTS

### 3.1. Effect of curcumin on neutrophil chemotactic activity

**Figure 1:**
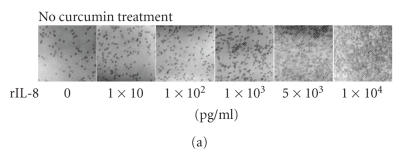

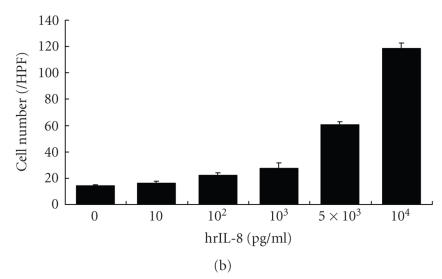

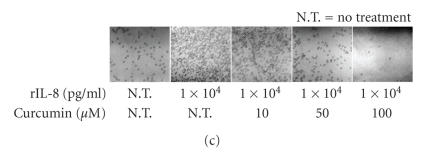

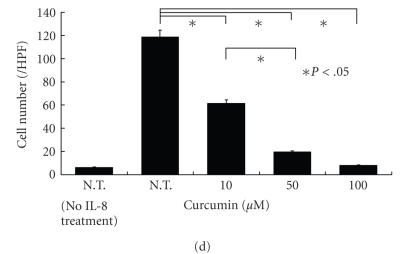
*The effect of curcumin on the chemotactic activity in human neutrophils*. Chemotaxis was assessed by using 24-well microchemotaxis chambers covered with membranes (3 *μ*m pore size). The lower wells were filled with a medium containing various concentrations of hrIL-8, and 1 × 10^6^ PMNs were then added to each well of the upper chamber. (a) The neutrophils that passed through the filter were counted. (b) hrIL-8 induced neutrophil chemotaxis in a dose-dependent manner. Next, the neutrophils were preincubated with various concentrations of curcumin for 2 hours at 37°C. Preincubated neutrophils were added to each well of the upper chamber, and the chemotaxis activity was assessed as described above. (c) Cells were counted after curcumin treatment. (d) Curcumin significantly inhibited the chemotactic activity induced by hrIL-8 at concentrations of 10–100 *μ*M in a dose-dependent manner. The neutrophil chemotactic activity determined in triplicate was expressed as the average number of migrating neutrophils/5 hpf. Each bar represents the mean ± SE of three individual experiments. Statistical significance versus the control: ∗*P* < .05.

Chemotaxis was assessed using microchemotaxis chambers covered with polyethylene terephthalate membranes. We observed that neutrophils rapidly passed through the membranes toward the wells of the lower chamber containing hrIL-8. After 1 hour of incubation, the neutrophils that passed through the filter to the lower wells were counted. In Figures 1(a) and 1(b) triplicate results of the average number of migrating neutrophils/5 hpf are shown. These data revealed that the chemotactic activity of neutrophils was stimulated by hrIL-8 in a dose-dependent manner.

To determine the effect of curcumin on neutrophil chemotaxis, the neutrophils were incubated with various concentrations of curcumin added to the wells of the upper chamber. As shown in Figures 1(c) and 1(d), curcumin significantly inhibited cell migration induced by hrIL-8 (10000 pg/mL) at concentrations of 10–100 *μ*M in a dose-dependent manner.

The obtained neutrophils were cultured in the presence of various doses of curcumin for 2 hours. The viability of neutrophils was examined using trypan blue staining. Curcumin did not affect neutrophil viability at a concentration up to 100 *μ*M when compared with the control treatment (data not shown). In these experiments, agonist stimulation at 37°C did not cause a significant decrease in the cell number or reduction in cell viability (>99% by trypan blue exclusion).

### 3.2. Curcumin inhibits the increase in the intracellular calcium concentration

To determine the effect of curcumin on intracellular signaling due to IL-8-mediated IL-8 receptor stimulation, we investigated the effects of changes in the intracellular calcium concentration.

**Figure 2:**
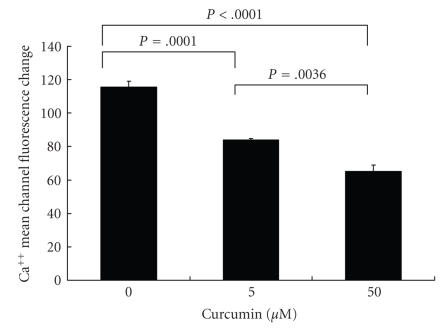
*Curcumin blocks the hrIL-8-induced intracellular* Ca^2+^ *enhancement*. Neutrophils (1 × 10^6^ cells/mL) treated with various concentrations of curcumin for 2 hours were loaded with 13 *μ*M Fura Red-AM in HEPES-buffered saline for 90 minutes at 37°C in a CO_2_ incubator. Addition of hrIL-8 (50 ng/mL) increased the intracellular calcium concentration and caused a decrease in the Fura Red fluorescence. The change in the fluorescence intensity at 640 nm was analyzed on an FACS calibur system. The mean channel of the fluorescence was analyzed by CellQuest software. The data obtained showed differences between the mean channel value before and after hrIL-8 stimulation. Curcumin significantly inhibited the increase in the intracellular calcium concentrations in a dose-dependent manner. Data was obtained from three independent experiments and was represented.

Approximately 1 × 10^6^ neutrophils treated with various concentrations of curcumin for 2 hours were loaded in a volume of 100 *μ*L in culture tubes containing 13 *μ*M Fura Red-AM in HEPES-buffered saline (135 mM NaCl, 4.6 mM KCl, 1.2 mM MgCl_2_, 11 mM HEPES, 11 mM glucose, 1.5 mM CaCl_2_ at pH 7.4) for 90 minutes at 37°C in a CO_2_ incubator. As shown in Figure 2, hrIL-8 (50 ng/mL) increased the intracellular calcium concentration, and this phenomenon was demonstrated as a decrease in the Fura Red fluorescence. Curcumin significantly inhibited the increase in the intracellular calcium concentrations in a dose-dependent manner. These data suggested that curcumin reduced neutrophil chemotaxis by affecting signal transduction mediated by IL-8 via the IL-8 receptors.

### 3.3. Curcumin promotes the downregulation of the expression of IL-8 receptors on the neutrophil surface

To investigate the status of IL-8 receptors after curcumin treatment, we examined the IL-8 receptor on the cell surface by FACScan using the CXCR1 and CXCR2 monoclonal antibodies.

**Figure 3:**
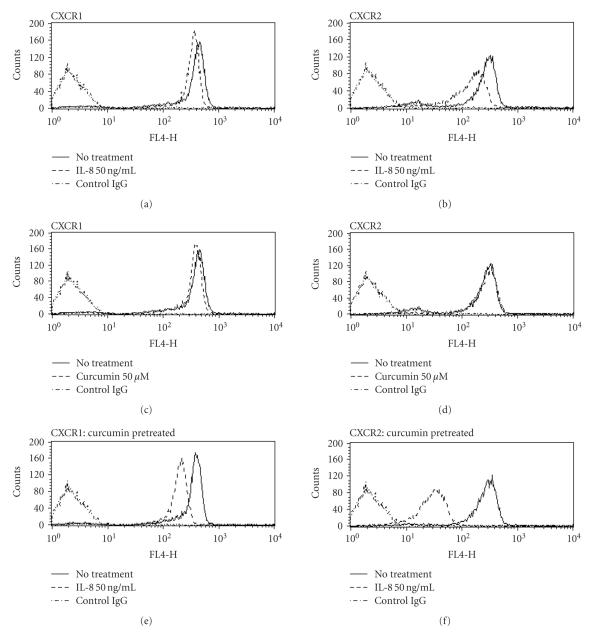
*Effect of curcumin on the internalization of the IL-8 receptor*. The obtained neutrophils were preincubated in medium with or without 50 *μ*M curcumin at 37°C for 2 hours. They were washed and incubated at 37°C for 2 hours with 50 ng/mL hrIL-8 diluted in BSA medium, while no hrIL-8 was added to the control tubes. The expressions of CXCR1 and CXCR2 present on the cell surface were determined with anti-CXCR1 ((a), (c), (e)) or anti-CXCR2 ((b), (d), (f)) mouse monoclonal antibodies by measuring the content of APC-conjugated anti-mouse IgG antibody using flow cytometry. CXCR1 (a) and CXCR2 (b) present on the surface of the untreated neutrophils were decreased by internalization after hrIL-8 (50 ng/mL) exposure. The amount of CXCR1 present on the cell surface slightly decreased after curcumin treatment (c), while that of CXCR2 shows a very slight change (d). A more dynamic decrease in the amount of CXCR1 (e) and CXCR2 (f) was observed in curcumin-treated neutrophils than in the untreated neutrophils after hrIL-8 treatment. Shown are representatives of three independent experiments with similar results.

As shown in Figure 3, both CXCR1 and CXCR2 present on the cell surface were downregulated by hrIL-8 (50 ng/mL) treatment due to receptor internalization. In addition, both IL-8 receptors present on the surface of neutrophils treated with curcumin were downregulated by rIL-8 (50 ng/mL) to a greater extent. These data revealed that the amounts of intracellular CXCR1 and CXCR2 are relatively increased by curcumin treatment. These findings indicated that the regulation of neutrophil chemotaxis may depend on the decreased amounts of CXCR1 and CXCR2 present on the cell surface.

### 3.4. Regulation of CXCR1 and CXCR2 by curcumin without de novo protein synthesis

To determine whether the production of IL-8 receptors in neutrophils has influenced the curcumin treatment, we also evaluated the change of IL-8 receptors without de novo synthesis which are transported to the cell surface.

**Figure 4:**
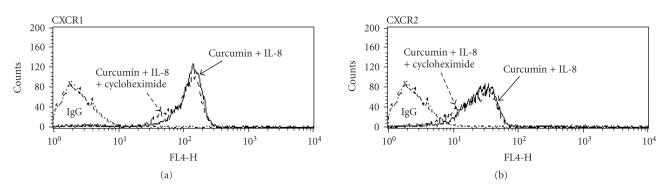
*Effect of curcumin on the reduction of the IL-8 receptors without protein synthesis*. After preincubation of neutrophils with curcumin (50 *μ*M) for 2 hours, they were incubated with hrIL-8 (50 ng/mL) for 30 minutes. While the neutrophils were incubating, cycloheximide (10 *μ*g/mL) was added to determine whether the de novo synthesis has any effect on the reduction of IL-8R by curcumin. The same decrease pattern of the amount of IL-8 receptor present on the cell surface was observed with cycloheximide. The inhibition of protein synthesis did not alter the reduction of both CXCR1 and CXCR2 by curcumin treatment. Shown are representatives of three independent experiments with similar results.

Neutrophils were incubated with cycloheximide, a strong protein synthesis inhibitor, at a concentration of 10 ng/mL. FACScan analysis showed the same decrease pattern of the amount of IL-8 receptor present on the cell surface. Cycloheximide treatment did not alter the reduction of IL-8 receptors after hrIL-8 treatment in curcumin-treated neutrophils (see Figure 4). These findings suggested that de novo synthesis of IL-8 receptors may not be associated with the changes due to curcumin treatment of IL-8 receptors present on the cell surface.

### 3.5. Effect of curcumin on the distribution of IL-8 receptors

To investigate the influence of curcumin on the degradation of IL-8 receptors, we determined the total cellular amount of IL-8 receptors by flow cytometry.

**Figure 5:**
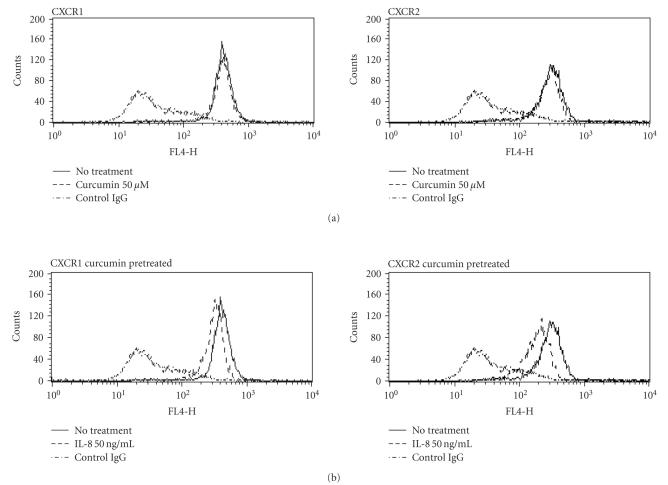
*The impact of curcumin on the total amount of IL-8 receptors*. Both IL-8 receptors on the cell surface and in the cytosol were stained using the permeabilization technique. The total cellular amount of the receptors was determined by flow cytometry. (a) The expression of total IL-8 receptors revealed no significant difference between curcumin-treated and untreated neutrophils. (b) Furthermore, in case of the curcumin-treated neutrophils, the total amount of IL-8 receptors showed a smaller decrease after IL-8 treatment than that shown by the receptors on the cell surface of curcumin-treated neutrophils (see Figures 3(e) and 3(f)).

The expression of total cellular amount of IL-8 receptors revealed no significant difference between the curcumin-treated and the control neutrophils (see Figure 5). However, in the curcumin-treated neutrophils, the IL-8 receptors on the cell surface showed obviously decrease than the control (see Figures 3(e) and 3(f)).

Taken together, these data suggested that curcumin changes the cellular distribution of IL-8 receptors. The increment of the intercellular IL-8 receptors may account for the decrement of IL-8 receptors on the cell surface after curcumin treatment.

### 3.6. Curcumin blocks CXCR recycling

**Figure 6:**
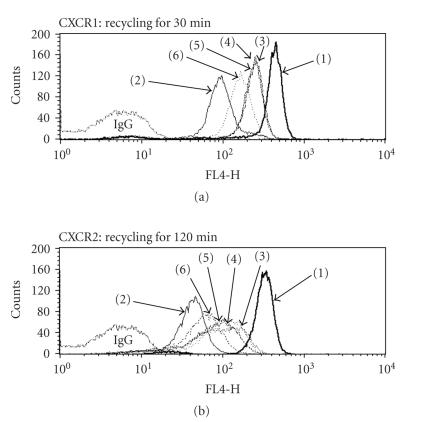
*Curcumin blocks the recovery process of IL-8 receptors*. The effect of curcumin on the recycling of IL-8 receptors to the surface of neutrophils was examined by FACScan. Internalization was induced by 30-minute exposure to 1000 ng/mL hrIL-8 at 37°C. In order to facilitate receptor recovery, the cells were washed once and resuspended in BSA medium containing 10 *μ*g/mL cycloheximide with various concentrations of curcumin at 37°C for the specified time periods. Following incubation, the cells were labeled as described above. (a) The cells were treated under the following conditions: (1) untreated cells; (2) cells treated with hrIL-8 and no recovery; (3) cells treated with hrIL-8 and recovery for 30 minutes; (4) cells treated with hrIL-8 and recovery for 30 minutes with curcumin (0.5 *μ*M); (5) cells treated with hrIL-8 and recovery for 30 minutes with curcumin (5 *μ*M); and (6) cells treated with hrIL-8 and recovery for 30 minutes with curcumin (50 *μ*M). (b) The cells were treated under the following conditions: (1) untreated cells; (2) cells treated with hrIL-8 and no recovery; (3) cells treated with hrIL-8 and recovery for 120 minutes; (4) cells treated with hrIL-8 and recovery for 120 minutes with curcumin (0.5 *μ*M); (5) cells treated with hrIL-8 and recovery for 120 minutes with curcumin (5 *μ*M); and (6) cells treated with hrIL-8 and recovery for 120 minutes with curcumin (50 *μ*M). Curcumin treatment blocked the recycling of CXCR1 (a) and CXCR2 (b) to the cell surface of the human primary neutrophils in a dose-dependent manner. Representative data is shown. Shown are representatives of three independent experiments with similar results.

In order to investigate the impact on the recycling pathway, the obtained neutrophils were pretreated with hrIL-8 (1000 ng/mL) at 37°C for 30 minutes. They were resuspended for the specified time periods in hrIL-8-free medium containing various concentrations of curcumin. Representative data of CXCR1 recycling for 30 minutes and CXCR2 recycling for 120 minutes are shown in Figure 6. The recycling of CXCR1 and CXCR2 to the cell membrane was blocked by curcumin in a dose-dependent manner (see Figure 6).

### 3.7. Rab11 interaction with CXCR

In order to assess the interaction of the small GTPase Rab11 that functionally regulates cytosolic trafficking of IL-8 receptors in human primary neutrophils, we analyzed anti-CXCR1 and anti-CXCR2 immunoprecipitates for the presence of Rab11.

**Figure 7:**
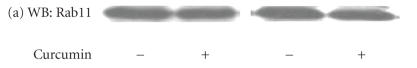

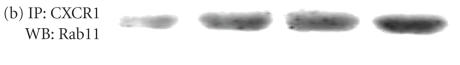

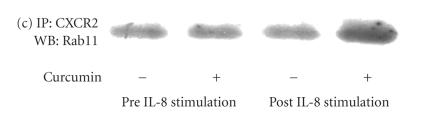

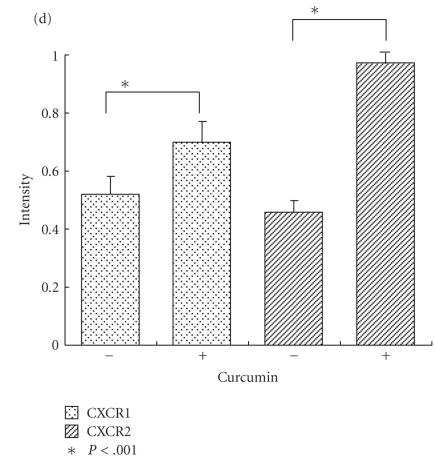
*Rab-11 interaction with CXCR*. (a) Proteins in the neutrophil lysates were separated by SDS-PAGE followed by blotting onto nitrocellulose membranes. The protein bands were detected by western blotting as described above. The amount of Rab11 protein was not remarkably different in curcumin-treated and untreated neutrophils. We analyzed anti-CXCR1 and CXCR2 immunoprecipitates for the presence of Rab-11. In primary neutrophils, analysis of the immunoprecipitates with anti-CXCR1 and anti-CXCR2 monoclonal antibodies supported the existence of an association with Rab-11. (b) Coimmunoprecipitation of Rab11 with CXCR1 revealed an increase after curcumin treatment. (c) After hrIL-8 exposure, coimmunoprecipitation of Rab11 with CXCR2 was enhanced though the association of Rab11 with CXCR2 not really appearing to be enhanced after curcumin treatment without IL-8 treatment. (d) In particular, curcumin-treated neutrophil showed remarkable increase ratio compared to the untreated neutrophil after IL-8 stimulation. Representative of three separate experiments are shown. The results of immunoprecipitates are expressed as an intensity ratio of the densitometry units relative to the amount of the basal Rab-11. Results are presented as the mean ± SD from three experiments.

Western blotting showed that the total amount of the Rab11 protein was not altered by curcumin treatment (see Figure 7(a)). Immunoprecipitation studies were performed to determine the association of CXCR1 and CXCR2 with Rab11 after curcumin treatment. Both receptors showed interaction with Rab11 (see Figures 7(b), 7(c), and 7(d)). These associations in both receptors were enhanced after curcumin treatment.

## 4. DISCUSSION

Various inflammations cause biological responses such as neutrophil activation through proinflammatory cytokine receptors; however, the mechanisms that control these cytokine receptors remain unclear. In this study, we reported that the chemotaxis of primary cultured human neutrophils was significantly inhibited in a dose-dependent manner by curcumin treatment. This inhibition did not result from curcumin-induced cell cytotoxicity, since the total number of cells and the cell viability at the end of the culture period did not differ with the culture conditions. Antichemotaxis provided important evidence for the anti-inflammatory responses due to curcumin treatment because neutrophil accumulation is a primary response in local inflammation, and this is followed by activation and infiltration of neutrophils.

Numerous chemokines play a crucial role in these biological responses. In particular, IL-8 is a strong neutrophil chemoattractant in the local inflammatory site. Other researchers, including our group, have already reported that curcumin inhibits the production of IL-8 by monocytes, macrophages, and other lymphatic cells [8]. The blockage of IL-8 production should be one of the key factors in the regulation of inflammation caused by neutrophils. Another way in which the inflammation elicited by neutrophils can be inhibited is by the interception of IL-8 promoted signal transduction in neutrophils.

In this study, neutrophils chemotaxis via hrIL-8 was significantly inhibited by curcumin. Experimental studies have demonstrated that human anti-CXCR monoclonal antibodies blocked IL-8-dependent cellular functions including neutrophil chemotaxis directly [34]. Our previous study reported that exogenous IL-8 could not recover response to IL-8 receptor by curcumin treatment [8]. Therefore, we examined about IL-8 signal transduction through IL-8 receptors after curcumin treatment.

In our study, a Ca^2+^ mobilization assay confirmed that IL-8 signal transduction through IL-8 receptors was blocked by curcumin treatment. Regarding the mechanisms of IL-8 receptor signaling, it is widely accepted that ligand-promoted internalization is one of the most important initiating pathways [19, 21, 22, 35]. In neutrophils, IL-8 stimulated the *β*-arrestin-dependent internalization of the CXCR receptor [19, 36]. Upon agonist binding, CXCR1 and CXCR2 receptor activation is followed by receptor phosphorylation at multiple serine residues and subsequent desensitization of the receptor to further stimulation [17, 21, 37]. These events are usually accompanied by receptor endocytosis and/or recycling of the receptor.

In our study, FACScan analysis showed a decrease in the amount of both CXCR1 and CXCR2 present on the cell surface after curcumin treatment. In particular, after IL-8 promoted receptor internalization, the amount of IL-8 receptors on the neutrophil cell surface is significantly decreased. Curcumin has been shown to inhibit NF-kB activity and to block a signal upstream of the NF-kB-inducing kinase and IKK and curcumin is suggested to regulate some transcriptional factors [9]. After blocking new protein synthesis by cycloheximide, our experiments showed the same decrease pattern in the IL-8 receptors present on the cell surface. This result suggests that the decrease in the amount of IL-8 receptors present on the cell membrane was not only due to degradation. The possibility of the receptor shedding should be considered, but there is no convincible report concerning the shedding of IL-8R. Moreover, Figure 5 demonstrated that the total amount of CXCR is not different after curcumin treatment, we consider that the possibility of the receptor shedding is deniable. This finding was consistent with those of our previous studies, suggesting that curcumin may affect the ligand-promoted trafficking pathway of the IL-8 receptor [8].

The trafficking pathway of IL-8 receptors is known to comprise two different transport directions including internalization and externalization. Either promotion of internalization or inhibition of externalization should induce a decrease in the amount of receptors present on the cell surface. Our experiments based on FACScan confirmed that curcumin delayed the trafficking pathway in the cytosol, and this resulted in the blockage of the recycling pathway to the cell surface. The Rab GTPase family is known to play an essential role in the cellular trafficking pathway. In particular, Rab11 is associated with the recycling compartment and trans-Golgi network (TGN) membranes, and it controls the slow endosomal recycling pathway as well as the traffic from the TGN [24, 26, 38]. The small GTPase Rab11 has been reported to regulate the trafficking of CXCR2 through the recycling endosome [27, 28]. Our immunoprecipitation study also showed the association of CXCR2 and Rab11 in primary neutrophils. Moreover, an association between CXCR1 and Rab11 was indicated. Further analyses are required to determine the mechanism of blockage of IL-8 receptor recycling; however, this is the first report on the association of the IL-8 receptor with Rab11 in primary cultured neutrophils. Following curcumin treatment, the binding of Rab11 and CXCR1 and CXCR2 appeared to be more prominent. The recycling process from the endosome to the cell surface remains unclear; however, stacking of the receptor proteins with the transporter proteins may be responsible for the deposition of IL-8 receptors in the endosomes. Therefore, it can be considered that these findings are consistent with those of previous studies that showed a reduction in the recycling of IL-8 receptors. Although our study had several limitations, these data suggest that curcumin might induce the stacking of the Rab11 vesicle complex with CXCR1 and CXCR2 in the endocytic pathway.

Moreover, curcumin is suggested to affect signal transduction from the other transmembrane receptors, such as transferrin receptor and the m4 muscarinic acetylcholine receptor, whose recycling is regulated by Rab11 through cytosolic trafficking pathways [39]. Since curcumin is also known to be an inhibitory factor for several serine/threonine kinases, such as PKC [40] and IKK [9], GTPases activity might be inhibited by kinase dephosphorylation, and this could result in an increased stability of the transporter protein association. Although phosphorylation is a crucial determinant of IL-8 receptor internalization, our western blotting data showed no significant difference in the mobility of the IL-8 receptor due to phosphorylation (data not shown). Previous studies were unable to completely explain the delays in CXCR1 and CXCR2 recycling to the cell surface by curcumin; however, it is possible that curcumin has an effect on the transport system due to recycling of the endosomes because the association of the IL-8 receptor with Rab11 after curcumin treatment was increased.

Recent studies suggested that curcumin has various mechanisms for bioactivity. These findings have identified curcumin as a regulator of kinase activities, a regulator of transcriptions, and a modulator of oxidative agent. Although there is no evidence about direct interact with membranous receptor, some investigators suggested that curcumin have a unique role in a modification of membranous or cytoskeleton system [41]. Previous study cannot fully explain about mechanisms of regulation of IL-8 receptor by curcumin, but our result is partially consistent with those of prior studies showing modification of cytosol components by curcumin [42].

As for our studies' limitation, we would have included an inactive analog in our study, if one had been available. There are several analogs and metabolites which have been reported [43], but almost analogs have a similar and modified effect of curcumin, which are not useful as an inactive control in this study.

In conclusion, our data provide some noteworthy evidence that curcumin inhibits neutrophil chemotactic activity caused by IL-8 chemokine. Curcumin is suggested to inhibit CXCR1 and CXCR2 recycling; this results in a decrease in neutrophil chemotaxis. In addition, we demonstrated that curcumin treatment changed the intercellular trafficking of the IL-8 receptor in neutrophils. These mechanisms may involve inhibition of the endosomal transport pathway by members of the Rab11 family.

Our study suggests that curcumin affects numerous bioactivities that involve signal transduction through the IL-8 receptor. Therefore, curcumin is able to function as a potent anti-inflammatory agent that regulates the receptor trafficking pathway in cytosol. In vivo use of curcumin might be beneficial for patients with inflammation due to the enhanced production of various proinflammatory cytokines, such as ARDS and pancreatitis as well as patients with the paraneoplastic syndrome. Recently a great deal of evidence has revealed that cytokine overreaction contributes to the severity of the systemic inflammation response syndrome (SIRS) [44]. Therefore, the control of cytokine signal transduction may play an important role in the inflammation therapy.

The unique mechanisms by which curcumin regulates the receptor transport system should be further elucidated.
